# Identifying influential determinants of women’s empowerment in Bangladesh using machine learning algorithms

**DOI:** 10.1371/journal.pone.0338037

**Published:** 2025-12-09

**Authors:** Md. A. Salam, Samiul Islam, Md. Mahfuz Uddin, Tamanna Rahman Shraboni, Antora Das, Md. Merajul Islam, Md. Rezaul Karim

**Affiliations:** 1 Department of Statistics, University of Rajshahi, Rajshahi, Bangladesh; 2 Department of Statistics, Jatiya Kabi Kazi Nazrul Islam University, Mymensingh, Bangladesh; Jahangirnagar University, BANGLADESH

## Abstract

**Background and objectives:**

Women’s empowerment is a vital issue in lower-middle-income developing countries like Bangladesh, where it plays a pivotal role in advancing development across the nation. Thus, this study aimed to identify the influential determinants of women’s empowerment in Bangladesh using machine learning (ML) algorithms.

**Materials and methods:**

The data for this study were obtained from the Bangladesh Demographic and Health Survey (BDHS) 2022, which included a nationally representative sample of 18,600 ever-married women aged 15–49 years. The important variables for women’s empowerment were identified using logistic regression and the Boruta feature selection method. Subsequently, eight popular machine learning algorithms - Decision Tree, Random Forest (RF), Naïve Bayes, Artificial Neural Network, Logistic Regression, Extreme Gradient Boosting, Gradient Boosting, and Support Vector Machine - were employed to predict women’s empowerment status. Model performance was assessed using accuracy, F_1_-score, and the area under the curve (AUC). Additionally, the most suitable model with SHAP analysis was used to identify the influential determinants driving women’s empowerment.

**Results:**

The RF-based model demonstrated the best performance, achieving an accuracy of 71.07%, an F_1_-score of 81.58%, and an AUC of 0.676. The analysis revealed age, division, wealth index, working status, household members, husband’s education, and respondent’s education as the most influential determinants of women’s empowerment.

**Conclusion:**

This study provides the best predictive model and identifies influential determinants of women’s empowerment in Bangladesh, offering valuable insights for achieving Sustainable Development Goal 5 (SDG-5) by 2030 through targeted actions and policies.

## 1. Introduction

Women’s empowerment is a fundamental pillar of global development, playing a critical role in raising sustainable economic growth, poverty reduction, and overall societal advancement [1,2]. It encompasses women’s active and equal participation in the financial, political, educational, and social spheres, ensuring they can contribute meaningfully to all aspects of life. Empowerment is about providing opportunities and creating an environment where women can exercise their rights, make informed decisions, and achieve personal growth. Women’s empowerment promotes gender equality, a prerequisite for achieving human rights and societal development. Empowering women with equal access to resources, education, healthcare, and economic opportunities allows them to overcome barriers and become active agents of change in their personal, family, and community lives. This participation drives positive outcomes such as improved family well-being, increased economic productivity, and stronger community resilience. Empowering women contributes to breaking cycles of poverty, as empowered women often invest in the health, education, and well-being of their children, fostering long-term intergenerational progress [3–5]. Women’s representation in political and decision-making processes also strengthens governance and leads to more inclusive and equitable policies.

Furthermore, women’s empowerment is directly linked to achieving key global development goals, such as the United Nations Sustainable Development Goals (SDGs), particularly Goal 5: Gender Equality [6]. Empowering women contributes to improved child health, reduced maternal mortality, enhanced education outcomes, and better economic resilience at both local and global levels. For instance, when women are educated and empowered, they are more likely to ensure their children receive education, improving the prospects of future generations. Women’s representation in political and decision-making processes also strengthens governance and leads to more inclusive and equitable policies. Overall, women’s empowerment is not merely a women’s issue—it is a societal imperative. It drives economic progress, strengthens communities, and ensures more inclusive, equitable, and sustainable development. It is therefore essential to identify the factors that affect women’s empowerment. Several studies have previously investigated these factors globally, including in Bangladesh [7–12]. However, most of these studies have used traditional statistical methods, such as logistic regression (LR), to identify the factors influencing women’s empowerment. LR assumes a linear relationship between predictors and the outcome variable, which may not always be appropriate when the data is more intricate or involves complex patterns. That’s why advanced methods like machine learning are essential. ML algorithms can handle large datasets and identify complex, non-linear relationships that traditional statistical methods may overlook. These algorithms can learn from data to uncover hidden patterns and interactions that could be crucial to understanding the factors influencing women’s empowerment. By examining the factors through robust methods, this research provides actionable insights to promote social transformation and help women attain autonomy and self-reliance in Bangladesh. However, no ML-based study was conducted to identify the factors influencing women’s empowerment in Bangladesh. Therefore, this study explores the application of ML algorithms to identify influential determinants of women’s empowerment in Bangladesh. The study’s findings highlight the significant role of ML in identifying and predicting the factors influencing women’s empowerment, providing valuable insights to guide policy decisions that promote gender equality and women’s empowerment in Bangladesh. By focusing on the influential determinants identified by the most suitable ML-based model, targeted interventions can be designed to further empower women. These interventions can contribute to achieving the Sustainable Development Goals (SDGs), particularly SDG 5, which emphasizes gender equality and women’s empowerment.

The remaining part of the study is organized as follows: Section 2 contains the materials and methods, including the data source, outcome variable, explanatory variables, statistical analysis, feature selection, machine learning algorithms, cross-validation, hyperparameter tuning, and model evaluation. Section 3 represents the analysis’s results. Section 4 discusses the findings, and Section 5 summarizes the conclusions.

## 2. Materials and methods

### 2.1. Data source

This study used the most recent Bangladesh Demographic and Health Survey (BDHS), 2022 data [13]. The data collection followed a two-stage stratified random sampling approach. In the first stage, 675 enumeration areas (EAs) were selected using probability proportional to size, including 438 rural and 227 urban areas. The survey was conducted among 30,375 residential households, including 10,665 in urban areas and 19,710 in rural areas. About 30,340 households from 30,375 households were interviewed, including ever-married women aged 15–49 years. After eliminating records with missing, unknown, or irrelevant information, 18,600 women were included in the final dataset for this study.

#### 2.1.1. Outcome variable.

The outcome variable of this study was women’s empowerment. Women’s empowerment was defined as involvement in either some or all of the three decisions: (1) their healthcare, (2) significant household purchases, and (3) visits to family or relatives [14]. It was coded as a binary variable, with empowerment categorized as Yes (1) if the woman participated in any of the decisions and No (0) if she did not participate in any of them.

#### 2.1.2. Explanatory variables.

We considered different types of explanatory variables, including demographic, socio-economic, environmental, and other factors, based on insights from previous studies and the availability of data in the BDHS, 2022 database [8,9,11,14]. The variables are respondent Age (15-19, 20-24, 25-29, 30-34, 35-39, 40-44, 45-49), Division (Barisal, Chittagong, Dhaka, Khulna, Mymensingh, Rajshahi, Rangpur, and Sylhet), Residence (urban, rural), Education (None, primary, secondary, and higher), Religion (Muslim, non-Muslim), Sex of the household head (male, female), Newspaper (not at all, < 1 week, and ≥ 1 week), Watching TV (not at all, < 1 week, and ≥ 1 week), Wealth index (poorest, poorer, middle, richer, and richest), Husband education (None, primary, secondary, and higher), Respondents currently working (no, yes), Number of household members (1–2, 3–4, 5 and more), Age at first-year marriage (10-14, 15-19, 20-24, and ≥25), Number of living children (0, 1–2, ≥ 3), and Wife-beating (no, yes). Wife beating was measured based on ‘agrees with at least one reason’ (agrees with husband is justified in hitting wife if she goes out without telling him, neglects the children, refuses sexual intercourse, or burns food).

#### 2.1.3. Statistical analysis.

Descriptive statistics of the study participants were reported as frequency (%) for the selected variables. The chi-square test was used to assess the bivariate associations between the response and explanatory variables, with a significance level of p-value < 0.05. Data analysis was carried out by using SPSS and the R programming language. The dataset was split into two sets: 80% for training and 20% for testing.

### 2.2. Feature selection

Feature selection is the process of selecting the most important features in a dataset by analyzing the underlying relationships within the data. This helps improve model performance, reduce complexity, and enhance interpretability [15]. In this study, we have applied LR and Boruta-based feature selection methods to select the most important features of women’s empowerment [16–21].

### 2.3. Machine learning algorithms

#### 2.3.1. Decision tree.

The Decision tree (DT) is a tree-like machine learning algorithm [16,22]. It splits a dataset into subsets based on the values of input features, forming a tree structure. Nodes represent tests on attributes (features), branches represent test outcomes, and leaves represent final outputs (class labels or values). The process starts with a root node for the entire dataset, which splits based on the most significant feature. Internal nodes refine the splits, while leaf nodes provide the final predictions. Internal nodes continue splitting the data, and leaf nodes provide the final predictions.

#### 2.3.2. Random forest.

Random forest (RF) is an ensemble ML algorithm that combines multiple DTs to enhance accuracy and reduce overfitting [23]. It uses bootstrap sampling (bagging) to create multiple subsets of the training data and uses random feature selection to ensure diversity among the trees. Each tree in the forest independently predicts an outcome, and the final prediction is made by majority voting [24].

#### 2.3.3. Naïve Bayes.

Naïve Bayes (NB) is a probabilistic ML algorithm based on Bayes’ theorem. It assumes that all features are independent of each other. Despite this “naïve” assumption, it performs well in many applications [25]. The algorithm calculates the probability of each class given the input features and predicts the class with the highest probability [26].

#### 2.3.4. Artificial neural network.

The Artificial neural network (ANN) is an ML algorithm inspired by the structure and functioning of the human brain [27]. The ANN consists of nodes organized into three main layers: input, hidden, and output. The data enters through the input layer, passes through one or more hidden layers where the neurons apply weights, biases, and activation functions, and finally produces an output through the output layer [28].

#### 2.3.5. Logistic regression.

Logistic regression (LR) is a widely used probabilistic classification model in ML. It assumes a linear relationship between the features and the log odds of the output variable [29]. During the training phase, LR estimates the model’s parameters using maximum likelihood estimation (MLE) to minimize the error in predicting class probabilities. Once trained, the model can predict the probability of the positive class by applying the logistic function to the input features. If the predicted probability exceeds a threshold (usually 0.5), the model classifies the input as the positive class; otherwise, it classifies it as the negative class.

#### 2.3.6. Extreme gradient boosting.

Extreme gradient boosting (XGBoost) is an optimized version of gradient boosting [30]. It builds decision trees as base learners in sequence, each trained to address the prediction error left by the preceding tree, leading to improved predictions. This sequential approach provides an alternative method for developing sophisticated, precise models with trees, enabling control over individual tree depth and complexity.

#### 2.3.7. Gradient boosting.

Gradient boosting is an ensemble ML method that builds a strong predictive model by sequentially combining multiple weak learners, typically DTs [31]. The process begins with a simple model, and in each subsequent step, new models are trained to predict the residual errors from the previous model. These new models are added to improve the overall prediction, with each model contributing to the final output based on a learning rate. The approach is guided by gradient descent to minimize the loss function.

#### 2.3.8. Support vector machine.

Support vector machine (SVM) is a widely used ML algorithm for classification and regression tasks [32]. It identifies the optimal hyperplane that maximizes the margin between classes in the dataset. SVM effectively handles linear and non-linear data using kernel functions, such as linear, polynomial, and radial basis function (RBF), transforming the data into higher-dimensional spaces [33]. This study employed the RBF kernel to achieve the largest possible margin between support vectors representing the closest data points from each class.

#### 2.3.9. Cross-validation and tuning hyperparameters.

Cross-validation (CV) is a popular protocol to assess a model’s performance more robustly. In K-fold CV, the original dataset is divided into K equal-sized subsamples or folds, where one of the K subsets is used as the validation or test set, while the remaining K-1 subsets are combined to form the training set. The models DT, RF, NB, ANN, LR, XGB, GB, and SVM contain hyperparameters that must be defined by the user prior to training to optimize model performance. Hyperparameter tuning was performed using a grid search combined with 10-fold (K = 10) cross-validation on the training dataset. For each fold, the data were split into training and test subsets at a 9:1 ratio. The caret package (version 6.0–93) in R was employed to identify optimal hyperparameter combinations for all eight models, as summarized in Table 1. In this study, we adopted a 10-fold CV protocol to assess a grid search for identifying the optimal hyperparameter value.

**Table 1:** Hyperparameter tuning ranges and criteria for eight ML-based models.

Model	Hyperparameters (Range/Values)	Tuning criterion
DT	cp=(0.001, 0.01, 0.05, 0.1); maxdepth=(5, 10,15, 20, 25); minsplit=(2, 5, 10, 20)	Maximum accuracy
RF	mtry=(1, 2, 3, 4, 5, 6, 7, 8, 9,10, 11,12, 13, 14)	Maximum accuracy
NB	fL=(0,0.5,1);usekernel=(TRUE, FALSE); adjust=(0.5,1,1.5,2)	Maximum accuracy
ANN	size=(1, 2, 3, 4, 5, 6, 7, 8, 9, 10); decay=(0, 0.1, 0.01, 0.001, 0.0001)	Minimizing loss
LR	C=(0.001, 0.01, 0.1, 1, 10, 100, 1000)	Maximum accuracy
XGB	nrounds=(100,500);max_depth=(10,15,20,25,30);colsample_bytree = seq(0.5, 0.9, length.out=5); eta = 0.1; gamma = 0; min_child_weight = 1;subsample = 1.	Minimizing loss
GB	n.tree=(100, 200, 300, 400, 500); interaction.depth=(1,3,5,7); shrinkage=(0.01, 0.05, 0.1); n.minobsinnode=(5,10,15)	Minimizing loss
SVM	cost=(0.1, 1, 10,100); gamma=(0.001, 0.01, 0.1, 1); kernel=(“linear”, “radial”)	Maximum accuracy

### 2.4. Model evaluation

Model evaluation is the process of assessing a machine learning model’s performance to ensure it generalizes effectively to unseen data [34]. We used the confusion matrix (Table 2), accuracy, sensitivity, specificity, ROC curve, and AUC to evaluate the models.

**Table 2:** Confusion matrix.

		Predictive Class	
		Positive (P)	Negative (N)
Actual Class	Positive (P)	True Positive (TP)	False Negative (FN)
	Negative (N)	False Positive (FP)	True Negative (TN)

**Accuracy:** The proportion of correctly predicted instances to the total instances.


$$Accuracy=\frac{TP+TN}{TP+TN+FN+FP}$$

**Sensitivity:** The proportion of actual positive cases correctly identified by the model.


$$Sensitivity=\frac{TP}{TP+FN}$$

**Specificity:** The proportion of actual negative cases correctly identified by the model.


$$Sensitivity=\frac{TN}{TN+FP}$$

#### 2.4.1. ROC curve.

The ROC curve is a graphical tool used to evaluate the performance of a binary classifier. It plots the true positive rate (sensitivity) against the false positive rate (1-specificity) across different threshold values, providing insight into how well the model can distinguish between positive and negative cases. The ROC curve helps assess the model’s diagnostic ability and the trade-offs between sensitivity and specificity [34].

#### 2.4.2. Model explain-ability.

The full form of SHAP is SHapley Additive exPlanations, first introduced by Lundberg and Lee (2017) to determine the contribution of each factor in a machine learning–based prediction model. It explains both local and global feature importance using SHAP values and is widely applied worldwide as an interpretability and visualization technique [35].

## 3. Results

### 3.1. Background characteristics of the study participants

Table 3 represents the bivariate association between sociodemographic characteristics and women’s empowerment. It was observed that younger women (15–19 years) showed lower empowerment (69.4%), while older women (35–44 years) demonstrated higher levels (89.2%−90.2%). Women living in the Sylhet division had the lowest empowerment (77.9%), whereas those in Dhaka and Rangpur had the highest (88.4% and 88.1%, respectively). Urban women have a higher empowerment rate (87.2%) than rural women (84.0%). Women with higher education showed greater empowerment (88.3%), whereas those with no education (85.8%), primary education (84.8%), and secondary education (84.2%) did not. Female-headed households report slightly higher (88.7%) compared to male-headed ones (84.6%). Women who read newspapers or watch TV more frequently exhibited higher empowerment (87.8%). The richest women were the most empowered (88.3%), while the poorest were the least (83.6%). Women with more educated husbands are slightly more empowered (86.4%). Working women report significantly higher empowerment (90.6%) than non-working women (82.8%). Women in smaller households (1–2 members) are more empowered (88.6%) than those in households with≥5 members (82.3%). Women who married later, at age ≥ 25, had the highest empowerment (91.1%), while those who married at ages 15–19 had the lowest (84.2%). Women with no children have the lowest empowerment (74.8%), while those with number of living children ≥ 3 are more empowered (87.5%). Women who oppose wife-beating showed higher empowerment (85.5%) than those who accept it (82.6%).

**Table 3:** Association between explanatory variables and women’s empowerment.

Explanatory variables	Overall n (%)	Empowerment status		p-value
		Yes, n (%)	No, n (%)	
**Age**				
15-19	1564(8.4)	1086(69.4)	478(30.6)	<0.001
20-24	3079(16.6)	2431(79.0)	648(21.0)	
25-29	3366(18.1)	2886(85.7)	480(14.3)	
30-34	3240(17.4)	2892(80.3)	348(10.7)	
35-39	3125(16.8)	2789(89.2)	336(10.8)	
40-44	2309(12.4)	2082(90.2)	227(9.8)	
45-49	2007(10.7)	1666(86.9)	251(13.1)	
**Division**				
Barisal	2006(10.8)	1658(82.7)	347(17.3)	<0.001
Chittagong	2760(14.8)	2418(87.6)	342(12.4)	
Dhaka	2787(15.0)	2465(88.4)	322(11.6)	
Khulna	2441(13.1)	1993(81.6)	448(18.4)	
Mymensingh	2019(10.9)	1706(84.5)	313(15.5)	
Rajshahi	2406(12.9)	2109(87.7)	297(12.3)	
Rangpur	2226(12.0)	1961(88.1)	265(11.9)	
Sylhet	1955(10.5)	1522(77.9)	433(22.1)	
**Residence**				
Urban	6484(34.9)	5654(87.2)	830(12.8)	<0.001
Rural	12116(65.1)	10178(84.0)	1938(16.0)	
**Education**				
None	2379(12.8)	2034(85.5)	345(14.5)	<0.001
Primary	4799(25.8)	4080(85.0)	719(15.0)	
Secondary	8618(46.3)	7242(84.0)	1376(16.0)	
Higher	2804(15.1)	2476(88.3)	328(11.3)	
**Religion**				
Muslim	16663(89.6)	14138(84.8)	2525(15.2)	0.002
Non-Muslim	1937(10.4)	1694(87.5)	243(12.5)	
**Sex of household head**				
Male	16344(87.9)	13831(84.6)	2513(15.4)	<0.001
Female	2256(12.1)	2001(88.7)	255(11.3)	
**Newspaper**				
Not at all	17280(92.9)	14671(84.9)	2609(15.1)	0.011
<1 week	786(4.2)	692(88.0)	94(12.0)	
≥ 1 week	534(2.9)	469(87.8)	65(12.2)	
**Watching TV**				
Not at all	8105(43.6)	6703(82.7)	1402(17.3)	<0.001
<1 week	1370(7.4)	1143(83.4)	227(16.6)	
≥ 1 week	9125(49.1)	7986(87.5)	1139(12.5)	
**Wealth index**				
Poorest	3302(17.8)	2761(83.6)	541(16.4)	<0.001
Poorer	3631(19.5)	3047(83.9)	584(16.1)	
Middle	3691(19.8)	3106(84.2)	585(15.8)	
Richer	3873(20.8)	3293(85.0)	580(15.0)	
Richest	4103(22.1)	3625(88.3)	478(11.7)	
**Husband education**				
None	3929(21.1)	3372(85.8)	557(14.2)	0.013
Primary	5210(28.0)	4420(84.8)	790(15.2)	
Secondary	5970(32.1)	5024(84.2)	946(15.8)	
Higher	3491(18.8)	3016(86.4)	475(13.6)	
**Currently working**				
No	12975(69.8)	10737(82.8)	2238(17.2)	<0.001
Yes	5625(30.2)	5095(90.6)	530(9.4)	
**Household member**				
1-2	977(5.3)	866(88.6)	111(11.4)	<0.001
3-4	7696(41.4)	6801(88.4)	895(11.6)	
5 and more	9927(53.4)	8165(82.3)	1762(17.7)	
**Age at first marriage**				
10-14	5067(27.2)	4358(86.0)	709(14.0)	<0.001
15-19	10477(56.3)	8818(84.2)	1659(15.8)	
20-24	2349(12.6)	2012(85.7)	337(14.3)	
≥25	707(3.8)	644(91.1)	63(8.9)	
**Number of living children**				
0	1955(10.5)	1462(74.8)	493(25.2)	<0.001
1-2	10965(59.0)	9400(85.7)	1565(14.3)	
≥3	5680(30.5)	4970(87.5)	710(12.5)	
**Wife beating**				
No	16159(86.9)	13816(85.5)	2343(14.5)	<0.001
Yes	2441(13.1)	2016(82.6)	425(17.4)	

### 3.2. Identified important features using logistic regression

Table 4 represents the results of logistic regression analysis. The results showed that women aged 15–19 (OR: 0.404, 95% CI: 0.323–0.506; *p* < 0.001) and 20–24 (OR: 0.570, 95% CI: 0.469–0.694; *p* < 0.001) years have lower odds of empowerment compared to those aged 45−49 years. In contrast, women in the aged group 40–44 years (OR: 1.314, 95% CI: 1.082–1.597; p = 0.006) had higher odds of empowerment. Women living in the Barisal (OR: 1.257, 95% CI: 1.064–1.489; *p* = 0.007), Chittagong (OR: 1.932, 95% CI: 1.642–2.272; p < 0.001), Dhaka (OR: 1.885, 95% CI: 1.594–2.229; p < 0.001), Mymensingh (OR: 1.469, 95% CI: 1.238–1.743; p < 0.001), Rajshahi (OR: 1.671, 95% CI: 1.400–1.994; p < 0.001) and Rangpur (OR: 1.889, 95% CI: 1.578–2.260; p < 0.001) were more likely to attain empowerment compared to those living in the Sylhet division. Women residing in urban areas (OR: 1.103, 95% CI: 0.999–1.218; p = 0.048) had higher odds of empowerment than those living in rural areas. Women who had educational level none (OR: 0.533, 95% CI: 0.425–0.669; p < 0.001), primary (OR: 0.616, 95% CI: 0.510–0.744; p < 0.001), secondary (OR: 0.734, 95% CI: 1.165–1.618; p < 0.001) had lower odds of being empowerment than higher educated women. Households headed by males (OR: 0.733, 95% CI: 0.635–0.846; p < 0.001) were less likely to have empowered women than those headed by females.

**Table 4:** Risk factor identification of women’s empowerment using logistic regression.

Explanatory variables	Categories	OR (95% CI)	p-value
Age	15-19	0.404(0.323-0.506)	<0.001
	20-24	0.57(0.469-0.694)	<0.001
	25-29	0.84(0.696-1.014)	0.070
	30-34	1.093(0.906-1.318)	0.354
	35-39	1.121(0.934-1.345)	0.221
	40-44	1.314(1.082-1.597)	0.006
	45-49^*^		
Division	Barisal	1.259(1.064-1.489)	0.007
	Chittagong	1.932(1.642-2.272)	<0.001
	Dhaka	1.885(1.594-2.229)	<0.001
	Khulna	1.061(0.902-1.246)	0.475
	Mymensingh	1.469(1.238-1.743)	<0.001
	Rajshahi	1.671(1.400-1.994)	<0.001
	Rangpur	1.889(1.578-2.260)	<0.001
	Sylhet^*^		
Residence	Urban	1.103(0.999-1.218)	0.048
	Rural^*^		
Education	None	0.533(0.425-0.669)	<0.001
	Primary	0.616(0.510-0.744)	<0.001
	Secondary	0.734(0.626-0.861)	<0.001
	Higher^*^		
Religion	Muslim	0.922(0.794-1.070)	0.285
	Non-Muslim^*^		
Sex of household head	Male	0.733(0.635-0.846)	<0.001
	Female^*^		
Newspaper	Not at all	1.256(0.947-1.665)	0.114
	<1 week	1.375(0.969-1.951)	0.075
	≥1 week^*^		
Television	Not at all	0.772(0.701-0.849)	<0.001
	<1 week	0.768(0.653-0.903)	0.001
	≥1 week^*^		
Wealth index	Poorest	0.92(0.775-1.093)	0.344
	Poorer	0.855(0.731-1.000)	0.050
	Middle	0.825(0.712-0.956)	0.011
	Richer	0.853(0.741-0.982)	0.027
	Richest^*^		
Husband education	None	1.072(0.897-1.280)	0.445
	Primary	1.103(0.943-1.290)	0.219
	Secondary	1.017(0.884-1.170)	0.811
	Higher^*^		
Respondent working	No	0.608(0.547-0.676)	<0.001
	Yes^*^		
Household member	1-2	1.581(1.268-1.971)	<0.001
	3-4	1.464(1.332-1.609)	<0.001
	5 and more^*^		
Age at 1^st^ marriage	10-14	0.834(0.622-1.119)	0.226
	15-19	0.808(0.610-1.070)	0.137
	20-24	0.754(0.563-1.010)	0.058
	≥25^*^		
Number of living children	0	0.691(0.573-0.832)	<0.001
	1-2	0.902(0.797-1.020)	0.101
	≥3^*^		
Wife beating	No	1.200(0.991-1.452)	0.052
	Yes^*^		

*: Indicates the reference category.

Women who watched TV either not at all (OR: 0.772, 95% CI: 0.701–0.849; p = 0.01) or < 1 time in a week (OR: 0.768, 95% CI: 0.653–0.903; p = 0.01) had lower odds of being empowered compared to those who watched TV ≥ 1 times in a week. Women in the middle (OR: 0.825, 95% CI: 0.712–0.956; p = 0.01) and richer (OR: 0.853, 95% CI: 0.741–0.982; p = 0.01) households had lower odds of empowerment than the richest women. Women with no working status (OR: 0.608, 95% CI: 0.547–0.676; p < 0.001) had lower odds of being empowered than their working counterparts. Women with 1−2 household members (OR: 1.581, 95% CI: 1.268–1.971; p < 0.001) and 3−4 household members (OR: 1.464, 95% CI: 1.332–1.609; p < 0.001) had greater chances of being empowered compared to those with ≥ 5 household members. Women with no live children (OR: 0.691, 95% CI: 0.573–0.832; p < 0.001) had lower odds of empowerment compared to those with ≥ 3 children. Women who had not been beaten by their husbands (OR: 1.279, 95% CI: 1.136–1.440; p < 0.001) were more likely to be empowered than those who had been beaten.

### 3.3. Identified important features using Boruta

**Fig 1:**
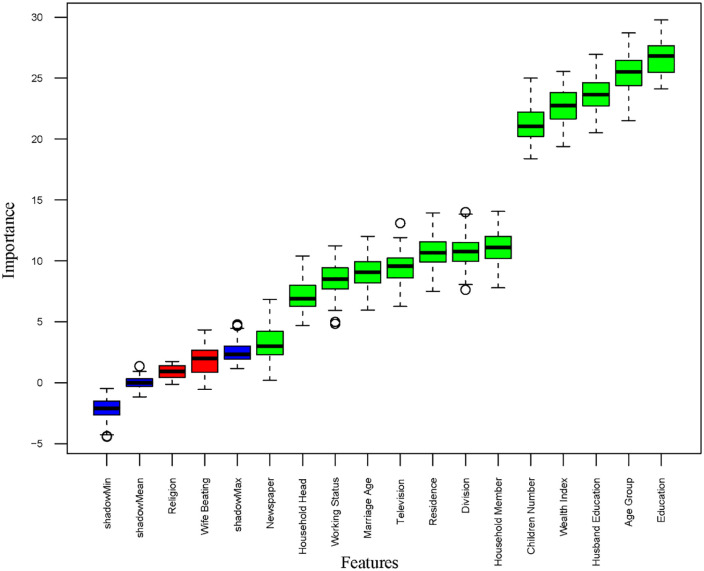
Important features for women’s empowerment.

Fig 1 displays the results of the Boruta-based feature selection method. The Boruta method showed that education, age, husband’s education, wealth index, number of living children, household members, division, residence, watching television, age at first marriage, currently working, sex of household head, and newspaper are important features for women’s empowerment. The variables deemed significant or important by either the LR or Boruta methods were utilized in the development of the ML-based models.

### 3.4. Performance comparison of ML models

**Fig 2:**
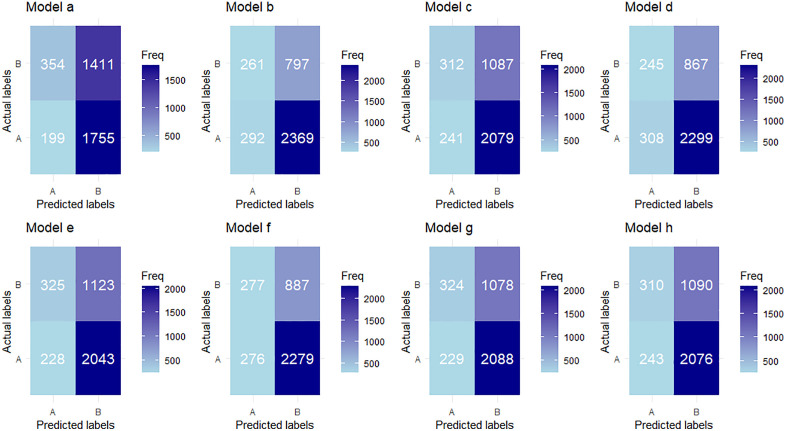
Confusion matrices of ML-based models.

Fig 2 shows the confusion matrices of ML-based models. In this figure, Model a represents DT, Model b represents RF, Model c represents NB, Model d represents ANN, Model e represents LR, Model f represents XGB, Model g represents GB, and Model h represents SVM.

**Fig 3:**
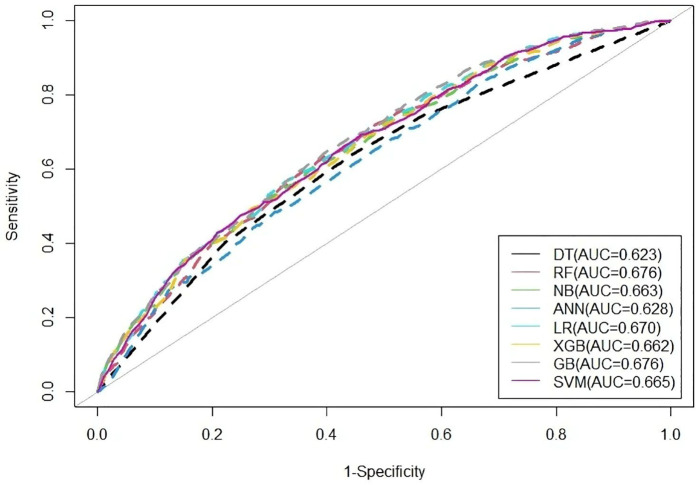
ROC curves of ML-based models.

Table 5 represents the prediction performance of different ML models. The results indicated that the RF-based model achieved the highest accuracy of 71.07%, specificity of 75.26%, precision of 89.98%, F1-score of 81.58%, and AUC of 0.676. In contrast, the DT-based model exhibited the highest specificity of 64.01%. All ML models were trained on 80% of the data and tested on the remaining 20%, with a random seed of 16160. The ROC curves of ML models, shown in Fig 3, revealed that the RF-based model had the largest area under the curve. Therefore, we proposed that the RF-based model is the best model for predicting women’s empowerment.

**Table 5:** Comparisons of the predicted performance of different ML models.

Models	Accuracy (%)	Sensitivity (%)	Specificity (%)	Precision (%)	AUC	F1-score (%)
DT	56.71	55.43	**64.01**	89.82	0.623	68.55
**RF**	**71.07**	**75.26**	47.12	**89.98**	**0.676**	**81.58**
NB	64.29	65.67	56.42	89.61	0.663	75.79
NN	68.41	72.61	44.30	88.19	0.628	79.65
LR	63.67	64.53	58.77	89.96	0.670	75.15
XGB	68.73	71.98	50.09	89.20	0.662	79.67
GB	64.86	65.95	58.59	89.90	0.676	76.16
SVM	64.16	65.57	56.06	89.52	0.665	75.70

Additionally, we computed uncertainty estimates by running each model across 5-, 10-, and 30-fold cross-validations to assess the robustness of our findings. The k-fold cross-validation results (Tables 6 and 7), which provide performance estimates across multiple runs, also demonstrate the superior performance of the RF model across 5-, 10-, and 30-fold settings. Based on these findings, the RF model is considered the most effective model for predicting empowered women in Bangladesh.

**Table 6:** Results of K-fold cross-validations of ML models based on accuracy.

Models	Accuracy(%) K-Folds			Mean	SD
	5-fold	10-Fold	30-Fold		
**DT**	56.71	56.71	56.71	56.71	0.00
**RF**	**71.06**	**71.07**	**71.74**	71.29	0.39
**NB**	65.15	64.29	65.15	64.86	0.50
**NN**	61.76	68.41	63.08	64.42	3.52
**LR**	63.65	63.67	63.65	63.66	0.01
**XGB**	67.36	68.73	67.49	67.86	0.76
**GB**	64.26	64.86	64.16	64.43	0.38
**SVM**	64.16	64.16	64.19	64.17	0.02

SD: Indicates Standard Deviation.

**Table 7:** Results of K-fold cross-validation of ML models based on F1-score.

Models	F1-Score (100%) K-Folds			Mean	SD
	5-fold	10-Fold	30-Fold		
**DT**	68.55	68.55	68.55	68.55	0.00
**RF**	**82.90**	**81.58**	**82.68**	82.39	0.71
**NB**	76.51	75.79	76.51	76.27	0.42
**NN**	73.46	79.65	74.75	75.95	3.27
**LR**	75.09	75.15	75.09	75.11	0.03
**XGB**	78.54	79.67	78.55	78.92	0.65
**GB**	75.63	76.16	75.62	75.80	0.31
**SVM**	75.12	75.70	76.15	75.66	0.52

**Fig 4:**
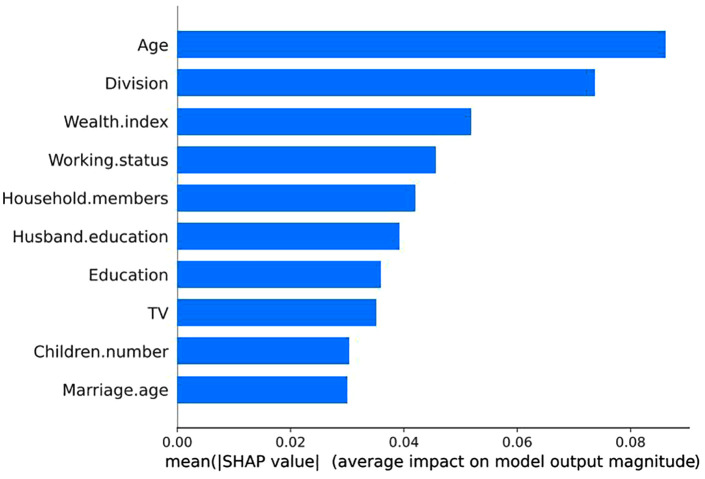
Importance of influential determinants based on mean absolute SHAP values.

**Fig 5:**
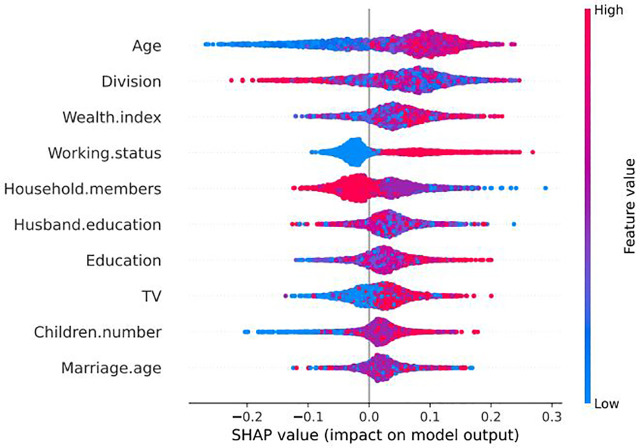
Importance of influential determinants based on local explanation summary.

The contributing predictors in the RF-based model for predicting women’s empowerment are presented in Fig 4 to explain global influential determinant importance and Fig 5 to display the direction of the relationship between a determinant and game outcome. These figures show that age group, division, wealth index, husband’s education, and working status are the top five features that influence women’s empowerment. These findings suggest that these five features are the most influential determinants of women’s empowerment in Bangladesh.

## 4. Discussion

This study investigates the use of ML algorithms to identify the influential determinants of women’s empowerment in Bangladesh, with the aim of supporting the achievement of SDG-5, which focuses on gender equality and the empowerment of women for sustainable development [36]. The study utilized the most recent BDHS, 2022 data to achieve this, which provides comprehensive socio-economic, health, and demographic information. Two popular feature selection methods were applied to identify the important features related to women’s empowerment. Subsequently, eight ML algorithms were used to build models using training data and to predict empowerment status on test data. The performance of each model was assessed using several metrics, including accuracy, sensitivity, specificity, precision,AUC, and F_1_-score. Among the models, the RF-based model achieved the highest performance regarding these evaluation metrics, making it the most effective model for predicting women’s empowerment status. Several previous studies have found that Random Forest (RF) was the most accurate and robust method for analyses based on BDHS or other datasets [37–39]. The superiority of RF can be attributed to: (i) its ensemble nature,(ii) Robustness to various feature types and outliers, (iii) Ability to capture nonlinear, interaction, and complex relationships, and (iv) Efficient handling of high-dimensional data with intrinsic feature selection. SHAP analysis was finally performed to identify the interpretable and influential predictors of women’s empowerment for the optimal prediction model (RF), based on SHAP values. The RF-based model with SHAP analysis revealed that age, division, wealth index, working status, husband’s education, and women’s education are the most influential determinants of women’s empowerment. To ensure the stability of our results, we conducted a robustness check by running each model with K-fold cross-validation for different values of K (K = 5, 10, 30). These results are provided in Tables 6 and 7. The results of Tables 6 and 7 confirm that the RF is robust for predicting women’s empowerment with the dataset.

Polin et al. [40] performed a study using ML-based algorithms to predict the impact of microcredit on women’s empowerment in rural areas of Bangladesh. To assess this impact, they collected data from three villages across three districts (Narayanganj, Kishoreganj, and Jessore). The dataset included 43 samples with 21 explanatory variables, focusing on women aged 18–50 years. They applied five machine learning algorithms—NB, k-Nearest Neighbors (k-NN), DT, RF, and Sequential Minimal Optimization (SMO)—to predict women’s empowerment status. Among these models, the DT-based approach achieved the highest accuracy of 83.75% and AUC 0.836. They demonstrated that microcredit positively influences women’s empowerment. Our findings differ somewhat from those of Polin et al. due to variations in objective, dataset, feature selection, geographical coverage, and timeframes.

Age is an influential determinant of women’s empowerment, and this finding aligns with previous studies [9–10,41]. They showed that women’s empowerment often increases with age as they gain more experience, education, and social influence. In younger years, women often focus on education and career development, which can enhance their autonomy and decision-making abilities. Additionally, social norms and expectations often change with age; younger women may experience societal pressures around marriage, appearance, and career choices, while older women may gain greater freedom from these expectations, enabling greater empowerment. However, as women age, especially during childbearing or caregiving years, they may face increased responsibilities that could limit their access to opportunities and independence, potentially impacting their empowerment. Furthermore, empowering older generations of women can positively affect younger women, as they benefit from stronger legal rights, better educational opportunities, and greater workforce participation, thereby enhancing their overall empowerment [9].

Divisional variation in women’s empowerment was observed, with women residing in the Chittagong, Rangpur, Dhaka, Rajshahi, Mymensingh, and Barisal divisions exhibiting higher levels of empowerment than those in Sylhet. Women in Chittagong, Rangpur, Dhaka, Rajshahi, Mymensingh, and Barisal divisions have higher empowerment rates than those in Sylhet, which can be attributed to a combination of education, economic opportunities, social norms, and infrastructure [36]. Education plays a crucial role, as higher female literacy and access to schooling contribute to greater awareness. These divisions have higher female literacy rates and more economic prospects, which help increase women’s participation in decision-making and financial independence [42]. In comparison, Sylhet has traditionally lagged in female education due to cultural norms and migration trends that prioritize male employment abroad over local female empowerment. Dhaka and Chittagong, as major urban centers, offer women more employment opportunities in industries such as garments, banking, and the service sector [43]. Rangpur and Mymensingh have seen active interventions from government and non-governmental organizations (NGOs) promoting women’s empowerment through microfinance, healthcare, and educational programs, which have been less prevalent in Sylhet. In contrast, Sylhet has a high rate of male outmigration, leading to an economy largely supported by remittances rather than local female labor participation. This reduces the necessity for women to seek employment, ultimately limiting their financial independence and empowerment. Dhaka and Chittagong benefit from a strong presence of government-led initiatives promoting women’s education and entrepreneurship. Cultural factors further influence the gap in women’s empowerment between these regions. Sylhet remains more conservative regarding gender roles, with societal norms often restricting women’s mobility and decision-making power. In contrast, Dhaka, Chittagong, Rangpur, Rajshahi, and Mymensingh have undergone gradual cultural shifts that support greater gender equality and encourage female participation in various sectors. Infrastructure and urbanization also contribute to the disparities. Dhaka and Chittagong offer better mobility, safety, and access to essential resources like education, healthcare, and financial services, which directly support women’s empowerment. Political engagement and legal awareness also vary across these divisions. In urban areas like Dhaka and Chittagong, women are more engaged in politics and local governance, allowing them to advocate for their rights and make informed decisions. Sylhet, however, has lower levels of female political participation and awareness of legal rights, which further hinders overall empowerment.

The wealth index is an influential determinant of women’s empowerment as it directly impacts their access to essential resources and opportunities [44]. Women from wealthier households are more likely to have access to quality education, which enables them to acquire the skills and knowledge necessary for greater independence and decision-making power. Financial resources also enable women to pursue economic opportunities, such as jobs or businesses, fostering economic independence and greater influence within the household and society [45]. Additionally, wealth improves access to healthcare services, contributing to better health and well-being, which is important for women to lead active and empowered lives. Wealthier women are also more likely to have greater social mobility, enabling them to participate in public life, challenge traditional gender norms, and advocate for their rights. Furthermore, access to technology and information, which is often more readily available to wealthier women, enhances their ability to connect with opportunities and engage in social and economic activities. Overall, the wealth index plays a central role in enhancing women’s autonomy, decision-making power, and overall empowerment. Working status is also an important determinant of women’s empowerment, as promoting women’s employment opportunities is a key policy aim for improving their general empowerment and socio-economic well-being. Education is an influential determinant of women’s empowerment, and this finding is corroborated by earlier studies [46,47]. The education of husbands and women plays a crucial role in shaping women’s empowerment, as it significantly influences a woman’s autonomy, decision-making ability, and access to resources. An educated husband is more likely to support his wife’s autonomy, including her education, career, and participation in community activities. This support fosters a more equal partnership, enabling women to have a voice in important family decisions, such as finances, healthcare, and child-rearing. Additionally, educated husbands tend to adopt more progressive attitudes toward gender roles, promoting gender equality within the household. Women’s education is perhaps the most direct factor influencing their empowerment. When women are educated, they gain the skills, knowledge, and confidence to make informed decisions about their own lives. Education enables women to enter the workforce, achieve financial independence, and gain a voice in family and community matters. It also helps them challenge traditional gender norms and fight against discriminatory practices. Educated women are more likely to be aware of their rights, demand better health services, and invest in their children’s education and well-being, creating a cycle of empowerment that can be passed on to future generations. Both husband’s and women’s education contribute to creating an environment of mutual respect, shared responsibilities, and equal opportunities, making them key determinants of women’s empowerment.

## 5. Conclusion

This study sought to explore the influential determinants of women’s empowerment in Bangladesh using ML-based algorithms. The study applied eight widely used ML algorithms to build predictive models, selecting variables using two feature selection techniques: LR and Boruta. The RF-based model outperformed the others in predictive performance, revealing that age, geographic division, wealth index, and both the husband’s and the woman’s educational levels are influential determinants of women’s empowerment in Bangladesh. These findings emphasize the importance of education, socio-economic status, and regional factors in empowering women in Bangladesh, offering valuable insights for targeted policy interventions to enhance women’s empowerment. By focusing on improving educational opportunities for women, addressing socio-economic disparities, and considering regional variations, policymakers can design more effective strategies to promote gender equality and empower women across the country.
